# A novel phage carrying capsule depolymerase effectively relieves pneumonia caused by multidrug-resistant *Klebsiella aerogenes*

**DOI:** 10.1186/s12929-023-00946-y

**Published:** 2023-08-31

**Authors:** Xiaohu Cui, Bing Du, Junxia Feng, Yanling Feng, Zheng Fan, Jinfeng Chen, Jinghua Cui, Lin Gan, Tongtong Fu, Ziyan Tian, Rui Zhang, Chao Yan, Hanqing Zhao, Wenjian Xu, Ziying Xu, Zihui Yu, Zanbo Ding, Zhoufei Li, Yujie Chen, Guanhua Xue, Jing Yuan

**Affiliations:** 1 Department of Bacteriology, Capital Institute of Pediatrics, Beijing, 100020 China; 2 Department of Clinical Laboratory, Children’s Hospital Affiliated to Capital Institute of Pediatrics, Beijing, China; 3 School of Biological Sciences, University of Edinburgh, Edinburgh, UK

**Keywords:** *Klebsiella aerogenes*, Pneumonia, Bacteriophage therapy, Depolymerase, Biofilms

## Abstract

**Background:**

*Klebsiella aerogenes* can cause ventilator-associated pneumonia by forming biofilms, and it is frequently associated with multidrug resistance. Phages are good antibiotic alternatives with unique advantages. There has been a lack of phage therapeutic explorations, kinetic studies, and interaction mechanism research targeting *K. aerogenes*.

**Methods:**

Plaque assay, transmission electron microscopy and whole-genome sequencing were used to determine the biology, morphology, and genomic characteristics of the phage. A mouse pneumonia model was constructed by intratracheal/endobronchial delivery of *K. aerogenes* to assess the therapeutic effect of phage in vivo. Bioinformatics analysis and a prokaryotic protein expression system were used to predict and identify a novel capsule depolymerase. Confocal laser scanning microscopy, *Galleria mellonella* larvae infection models and other experiments were performed to clarify the function of the capsule depolymerase.

**Results:**

A novel lytic phage (pK4-26) was isolated from hospital sewage. It was typical of the *Podoviridae* family and exhibited serotype specificity, high lytic activity, and high environmental adaptability. The whole genome is 40,234 bp in length and contains 49 coding domain sequences. Genomic data show that the phage does not carry antibiotic resistance, virulence, or lysogenic genes. The phage effectively lysed *K. aerogenes *in vivo, reducing mortality and alleviating pneumonia without promoting obvious side effects. A novel phage-derived depolymerase was predicted and proven to be able to digest the capsule, remove biofilms, reduce bacterial virulence, and sensitize the bacteria to serum killing.

**Conclusions:**

The phage pK4-26 is a good antibiotic alternative and can effectively relieve pneumonia caused by multidrug-resistant *K. aerogenes*. It carries a depolymerase that removes biofilms, reduces virulence, and improves intrinsic immune sensitivity.

**Supplementary Information:**

The online version contains supplementary material available at 10.1186/s12929-023-00946-y.

## Introduction

*Klebsiella aerogenes,* a common gram-negative, facultative anaerobic bacterium belonging to the family Enterobacteriaceae, has been reported to be an important opportunistic and multidrug resistant (MDR) pathogen in humans, particularly patients who are on mechanical ventilation [1]. From the early 1990s to 2003, *K. aerogenes* was the most clinically prevalent cause of nosocomial *Enterobacter* infections, and MDR *K. aerogenes* was an increasing cause of hospital-acquired infections [2]. In addition to its intrinsic resistance to ampicillin [3], *K. aerogenes* also has a broad ability to develop antibiotic resistance mechanisms [4, 5]. Moreover, there have been reports of carbapenem-resistant *K. aerogenes* with polymyxin resistance [6, 7], which may lead to the emergence of untreatable invasive infections.

*Klebsiella aerogenes* can form biofilms in endotracheal tubes and cause ventilator-associated pneumonia in mechanically ventilated patients [8, 9]. *K. aerogenes* strains with thicker capsules have higher antimicrobial resistance, stronger phagocytosis resistance, and lower serum sensitivity [10, 11]. There is a lack of effective and safe treatment options to control MDR *K. aerogenes* infection; therefore, developing alternatives to antibiotics to control colonization, remove established biofilms and reduce virulence is urgent and important.

As natural predators of bacteria, phages have the potential to address the absence of drugs in the post-antibiotic era [12]. Phages are host-specific and can effectively lyse bacteria. In recent years, phage therapy has been used to treat diseases caused by MDR pathogens, such as lung infections in transplant patients and bone and joint infections [13, 14]. In addition to the direct use of phages, other antimicrobial methods associated with phages include the use of endolysin and depolymerases [15]. Depolymerases can bind and specifically cleave polysaccharide-repeating units of host bacteria and destroy biofilms while antibiotics lack these abilities [16–18]. Compared with phages, the effect of on bacteria is more stable, consistent across strains with the same capsule type, and depolymerases have higher thermal stability, making them suitable for mass production [19]. In recent years, several studies have reported on phages that target *K. aerogenes*, but there has been a lack of phage therapeutic explorations, kinetic studies, and depolymerase mining.

In this study, a novel lytic phage (pK4-26) was isolated from hospital sewage, and its biology and genomic characteristics were determined. It can effectively lyse *K. aerogenes *in vivo, slow inflammation progression, and reduce mortality in mouse pneumonia models. Moreover, a novel phage-derived depolymerase (*K4-26 dep* protein) was predicted and indicated to be able to digest the capsule and degrade biofilms. After co-incubation with *K4-26 dep* protein, the bacteria showed lower virulence and higher innate immune sensitivity. This phage and the depolymerase it carries have the potential to be used in future antimicrobial therapy.

## Methods

### Bacterial strains, cells, and mice

All strains were isolated from clinical samples [20]. All strains were incubated in Luria–Bertani (LB) medium (Oxoid Ltd., UK) at 37 °C. An MDR strain (*K. aerogenes* 4-26) isolated from the endotracheal tube tip was used as a host for the isolation and proliferation of phage. Human lung carcinoma A549 cells were cultured in Dulbecco’s modified Eagle’s medium containing 10% fetal calf serum at 37 °C with 5% CO_2_. Male C57 BL/6J mice (7–8 weeks old) were purchased from the Beijing Vital River Laboratory Animal Technology Co., Ltd. (Beijing, China).

### Isolation and biological characterization of phage pK4-26

Phage was isolated from organic sewage water in the Children’s Hospital Affiliated to Capital Institute of Pediatrics using the double-layer overlay technique. Phage titers were measured using a double agar overlay plaque assay. Different phage concentrations were mixed with log-phase host stains and co-culture for 3 h at 37 °C to measure the optimal multiplicity of infection (MOI). The host strain in log-phase was mixed with phage particles at the optimal MOI and incubated at 37 °C and the phage titers were calculated every 10 min over 150 min to generate a one-step growth curve. The temperature and pH stability of pK4-26 was determined by incubating phage preparations at different temperatures or pH values. After treatment, titers of residual phages were counted by double-layer plaque assay. The host ranges were examined using a standard spot test and an efficiency of plating (EOP) assay. The phage morphology was observed using transmission electron microscopy (TEM) operated at 80 kV.

### *Klebsiella aerogenes* infection and phage therapy in mouse models

The minimum lethal dose (MLD)was determined, and the animal model was constructed according to previous reports [21, 22]. Briefly, the mice were randomly allocated into four groups: (1) the *K. aerogenes* group (n = 25), which received an intratracheal/endobronchial instillation of 2 × 10^7^ CFU *K. aerogenes* (2 × MLD) and intranasal delivery of normal saline; (2) the *K. aerogenes* + pK4-26 group (n = 25), which received an intratracheal/endobronchial instillation of 2 × 10^7^ CFU *K. aerogenes* and intranasal delivery of 2 × 10^9^ PFU pK4-26; (3) the pK4-26 group (n = 25), which received an intratracheal/endobronchial instillation of normal saline and intranasal delivery of 2 × 10^9^ PFU pK4-26; and (4) the paired group (n = 25), which received an intratracheal/endobronchial instillation of normal saline and intranasal delivery of normal saline. Three mice were euthanized to monitor bacterial loads and phage titers in the lungs at 2 h, 6 h, 12 h, 24 h, 48 h, 72 h and 7 days post infection. The lung tissues were also used for cytokine (IL-1β, IL-6, and TNF-α) level determination, and the lung, liver, kidney, and spleen tissues were used for hematoxylin–eosin staining at 24 h and 48 h post infection.

### Genome sequencing and analysis

The genomic DNA of the phage was extracted using the phenol–chloroform method as previously described [23]. Whole-genome sequencing of DNA was performed on the Illumina HiSeq 2500 platform (Berry Genomics Corp., China). Potential coding domain sequences (CDS) were identified using PHASER [24], and putative homologies with predicted phage proteins were identified using BLASTP. A genomic map was constructed using the CGView Server. Phylogenetic trees based on amino acid sequences of the large terminase subunit, and the putative depolymerase gene sequences were constructed by MEGA 11, using the maximum likelihood method with 1000 bootstrap replicates. The resulting trees were plotted, annotated, and visualized using ggtree v3.2.1 [25]. Genome comparisons of pK4-26 and other phages were performed using Easyfig 2.2.5.

### Protein domain and structure prediction

Protein domain prediction of the putative depolymerase was performed using BLASTP. The protein sequences of phages hosted by *K. aerogenes* were downloaded from the National Center for Biotechnology Information (NCBI) viral genome database and analyzed using PfamScan. Protein structure prediction of the putative depolymerase was performed using the Robetta server, and the predicted structure was visualized in PyMol2.4.

### Expression and purification of putative depolymerase

The CDS (including stop codons) was inserted into the pET-28a expression vector via the *Bam*HI and *Xho*I restriction sites. The primers and the cycling program are described in Additional file 3: Table S1. The recombinant plasmids were transformed into Escherichia coli BL21 cells and induced by adding 0.1 mM IPTG and incubating at 4 °C overnight. The resulting His-tagged protein was purified using Ni Sepharose 6FF (Solarbio, Beijing, China). The protein concentration was determined by the bicinchoninic acid method.

### Determination of anti-capsule ability

Different concentrations of purified recombinant depolymerase were spotted into a lawn of the host strain to test its host range. TEM was used to observe the morphological changes in *K. aerogenes* 4-26 after incubation with 100 ng/μL of depolymerase solution at 37 °C for 6 h. The CPSs were extracted using a phenol-extraction method according to a previous method [26]. The capsular polysaccharides (CPSs) were incubated with different concentrations of the depolymerase for 12 h at 37 °C to verify their anticapsule ability. After incubation, the concentration of reducing sugars in each tube was determined using a 3,5-dinitrosalicylic acid (DNS) test kit (Solarbio, Beijing, China).

### Formation, observation, and quantitation of biofilms

Bacteria cultured overnight were diluted 100-fold, transferred onto confocal dishes, and incubated at 37 °C for 24 h to form biofilms. After removing the supernatants and washing confocal dishes three times using PBS, different concentrations of depolymerase were added and incubated for 24 h at 37 °C. The formed biofilms were fixed using 4% glutaraldehyde for 3 h, stained with 50 μg/mL FITC-ConA for 20 min and then stained with 5 μg/mL PI for 15 min. The stained biofilms were visualized by confocal laser scanning microscopy (Leica TCS SP8 X, Germany).

Quantitation of biofilms was performed using a crystal violet staining method. The same bacteria as the above treatment were transferred onto 96-well flat-bottomed microtiter plates and incubated for 24 h at 37 °C. After removing the supernatants and washing the wells three times, 200 μL of PBS or different concentrations of depolymerase were added to the wells and incubated for 6, 12 and 24 h at 37 °C. After removing the supernatants and washing using with PBS, the wells were stained with a 0.75% crystal violet solution for 30 min and washed twice with purified water. The wells’ contents were solubilized in 200 µL of anhydrous ethanol for 30 min, and the optical density at 570 nm (OD_570_) was measured to quantify of the biofilms.

### Bacterial virulence determination

A549 cells (4 × 10^5^) were cultured in a 96-well plate and infected with different concentrations of *K. aerogenes* 4-26 for 3 h, which were pretreated with 100 ng/μL of depolymerase solution at 37 °C for 6 h or PBS. The number of viable cells was determined using the cell counting kit‐8 (CCK‐8) method (Beyotime Biotechnology Co., Shanghai, China). *G. mellonella* larvae infection models were also used to compare the virulence of bacteria. Briefly, different concentrations of bacterial or normal saline was injected into the last pair of pro-left legs. Then the larvae were incubated at 37 °C. Each group contained ten larvae. The time of death of each larva was recorded.

### Serum killing assay

The bacteria were pretreated with depolymerase or PBS using the same method as above. Then, the bacteria were mixed with human serum from healthy volunteers and incubated at 37 °C for 6 h. The percent survival of bacteria was calculated based on viable counts relative to the initial inoculum. Three replicates were set up for each experimental condition.

### Thermal and pH stability of depolymerase

The temperature and pH stability of depolymerase was determined by incubating depolymerase preparations (100 ng/μL) at different temperatures or pH values. After treatment, the preparations were incubated with equal amounts of CPS solution at 37 °C for 6 h. The concentrations of reducing sugars were determined using a DNS test kit. The percent survival of depolymerase was calculated based on the concentration of reducing sugars relative to the highest sugar concentration.

### Statistical analysis

Data are presented as the mean ± SD and were analyzed with one-way ANOVA using SPSS 20.0 (IBM Corp., USA). Groups were compared with Kruskal–Wallis test for survival function. Differences were judged statistically significant at a *P* value of < 0.05.

## Results

### A novel lytic *Podoviridae* phage hosted by *K. aerogenes* was isolated

**Fig. 1:**
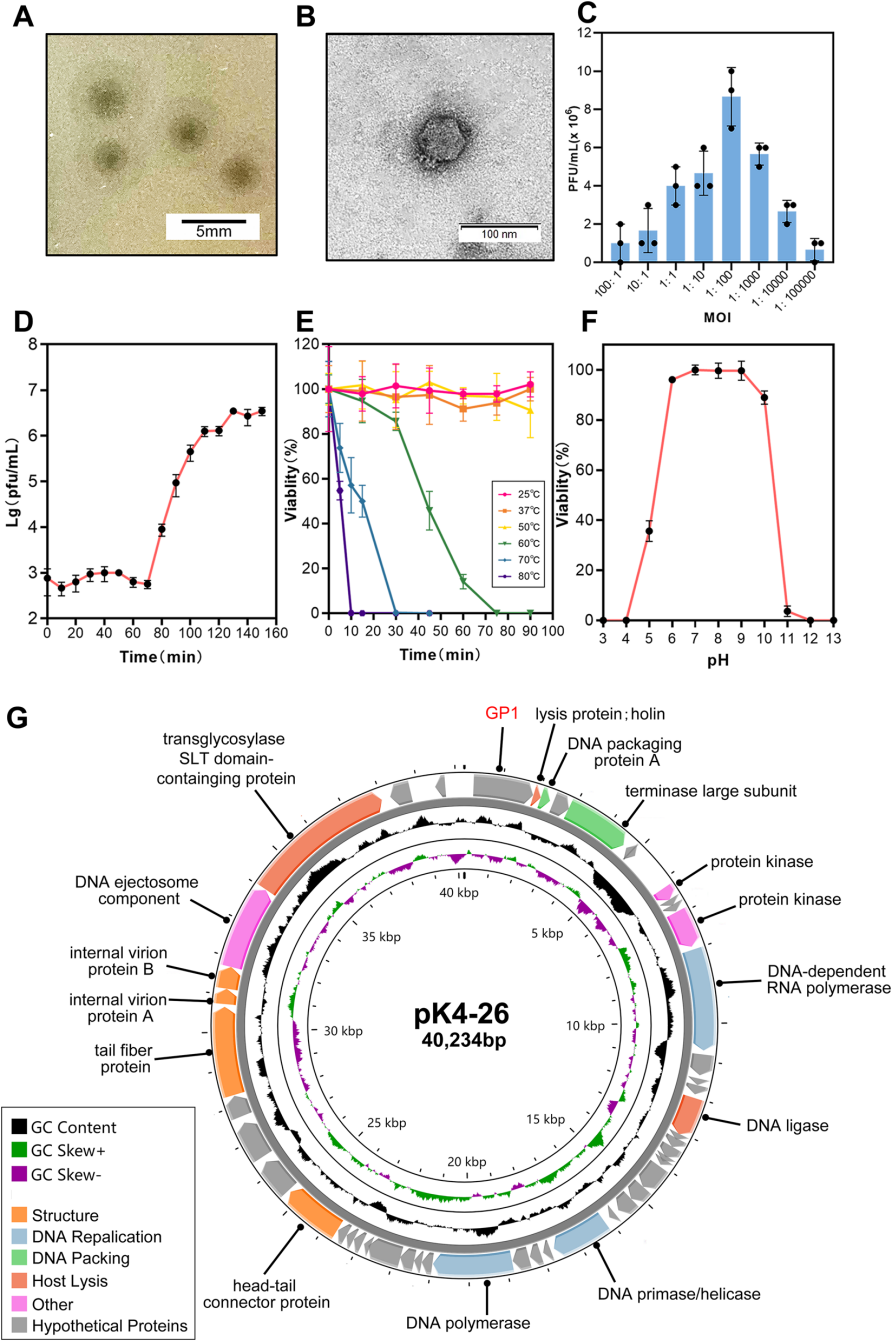
Morphology, biological characterization, and genomic features of phage pK4-26. **A** Phage plaques of pK4-26. **B** Phage morphology observed by TEM. **C** Optimal multiplicity of infection of phage. **D** One-step growth curve. **E** Thermal stability of pK4-26. **F** pH stability of pK4-26. **G** The genomic map of pK4-26. Values are expressed as mean ± SD (n = 3)

A bacterial strain (*K. aerogenes* 4-26) was isolated from the tip of a tracheal tube, and the strain showed multidrug resistance (Additional file 3: Table S2). A lytic phage named pK4-26 was isolated from hospital sewage using the strain as a host. The phage formed clear plaques with halos in double-layer agar plates, and the diameter of the plaques was 3.03 ± 0.34 mm (Fig. 1A). The phage was typical of the *Podoviridae* family, possessing an isometric head with a diameter of approximately 45 nm and short tails (Fig. 1B). It infected 6 of the 13 *K. aerogenes* strains in our laboratory (Table 1). The optimal MOI was 0.01, and a one-step growth curve showed that the latent period was 70 min, and the average burst size was 34.7 PFU/cell (Fig. 1C, D). The phage remained stable in environments below 50 °C and at pH values between 6 and 10 (Fig. 1E, F).

**Table 1:** The host range of phage and depolymerase

Genus	Isolation	Source	Serotyping/ST	EOP	Depolymerase
*K. aerogenes*	4-26	Endotracheal tube	PSgc4	1	+
*K. aerogenes*	415	Fecal	PSgc4	1	+
*K. aerogenes*	411	Fecal	PSgc4	1	+
*K. aerogenes*	424	Fecal	PSgc4	1	+
*K. aerogenes*	425	Fecal	PSgc4	1	+
*K. aerogenes*	LB-1	Fecal	PSgc4	1	+
*K. aerogenes*	4141	Urine	PSgc1	−	−
*K. aerogenes*	4150	Ascites	PSgc1	−	−
*K. aerogenes*	4221	Fecal	PSgc1	−	−
*K. aerogenes*	4171	Endotracheal tube	PSgc1	−	−
*K. aerogenes*	2198	Blood	PSgc1	−	−
*K. aerogenes*	4534	Urine	Unknown	−	−
*K. aerogenes*	ATCC 13048	Reference strain	PSgc1	−	−
*K. pneumoniae*	Reference [20]	Reference [20]	–	−	−

The genome of phage pK4-26 is 40,234 bp in length and there are 49 CDSs. The complete genome sequences of pK4-26 are most similar to *Klebsiella* phage KN3-1 (NC_048131.1), and the identity and query coverage between the two phages were 85.77% and 80%, respectively (Additional file 3: Table S3), which verified that pK4-26 is a novel phage. Furthermore, the genomic data show that pK4-26 carries no antibiotic resistance, virulence, or lysogenic genes (Fig. 1G, Additional file 3: Table S4). Therefore, this novel phage is suitable for phage therapy.

### Phage pK4-26 can effectively lyse *K. aerogenes* in vivo and relieve pneumonia

**Fig. 2:**
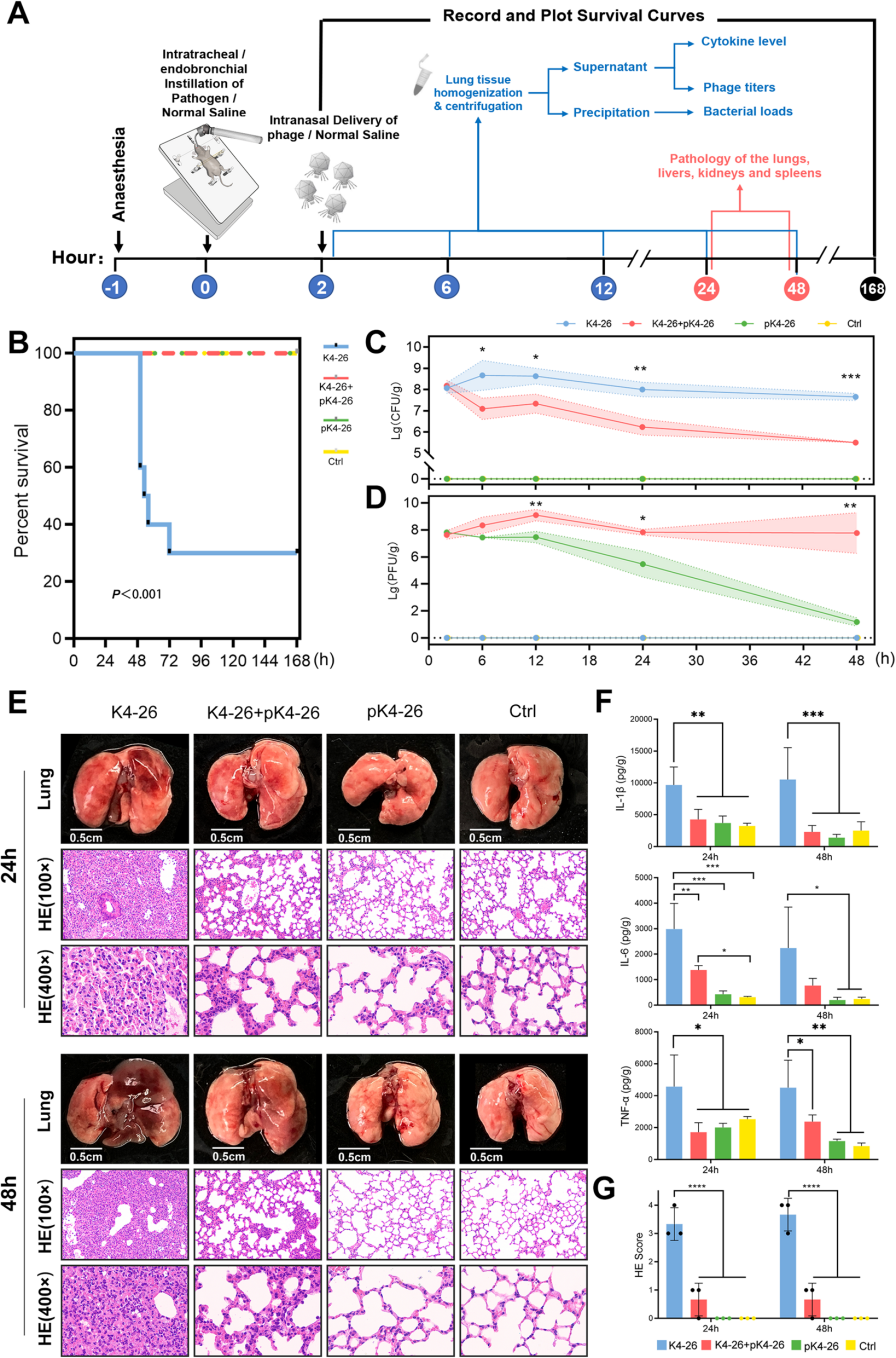
Phage pK4-26 can effectively lyse *K. aerogenes *in vivo and relieve pneumonia. **A** Schematic diagram of the experimental procedure for phage therapy. **B** Survival of mice infected with *K. aerogenes* followed by phage therapy (Kruskal–Wallis’s test). **C** Changes in bacterial loads in the lungs were recorded for 48 h. **D** Changes in phage titers in the lungs were recorded for 48 h. **E** Anatomy, H&E staining (×100 and ×400 magnifications) of lungs sections from each group for 24 and 48 h. **F** Proinflammatory cytokine levels in the lungs at 24 and 48 h. **G** Histopathological scores of lung tissue sections. The histopathological score was related to the following distribution: 1, thickened alveolar walls; 2, edema; and 3, tissue parenchymatous lesions such as congestion and hemorrhage. Tissue sections were evaluated by two trained pathologists with the following scores: 0, no pathological lesion; 1, mild; 2, moderate; and 3, severe. Values are expressed as means ± the SD (n = 3 mice/group). Values are expressed as mean ± SD (n = 3). *P < 0.05; **P < 0.01; ****P < 0.0001 (one-way ANOVA)

To assess the therapeutic effect of pK4-26 in vivo, a mouse pneumonia model was constructed by intratracheal/endobronchial delivery of *K. aerogenes* 4-26 (Fig. 2A). We found that 2 × 10^7^ CFU *K. aerogenes* 4-26 caused death in 70% of mice in 48 h to 7 days. However, intranasal administration of pK4-26 was effective in preventing mouse mortality (Fig. 2B). When the bacteria were delivered into the lungs of the mice, they grew rapidly and stabilized at 10^7^–10^8^ CFU/g. After phage treatment, the bacteria decreased rapidly by approximately 141-fold within 48 h while the phage titers increased 19-fold in 12 h. Phage titers in the phage therapy group stabilized at 10^7^–10^8^ PFU/g in 48 h while almost no phages were left in the group treated with pK4-26 alone (Fig. 2C, D), which suggests that pK4-26 can rapidly lyse bacteria and replicate itself in vivo.

*Klebsiella aerogenes* 4-26 caused lung congestion, distension, and hepatomegaly in mice within 24 h and lung necrosis by 48 h. Pathological staining showed collapsed alveolar structures, and diffuse neutrophil infiltration with scattered hemorrhaging and necrosis (Fig. 2E). Cytokines in the lungs were correspondingly elevated (Fig. 2F). Phage treatment prevented progressive necrosis in the lungs of mice and reduced lung cytokine levels; pathological staining also showed only slight alveolar wall thickening in the phage therapy group. More importantly, phage treatment did not cause damage to other organs, nor did it cause elevated cytokines (Fig. 2E, F, Additional file 1: Fig. S1). No resistant bacteria were detected during phage treatment (Additional file 2: Fig. S2).

### Unique characteristics of the phage were discovered by comparative genomics

**Fig. 3:**
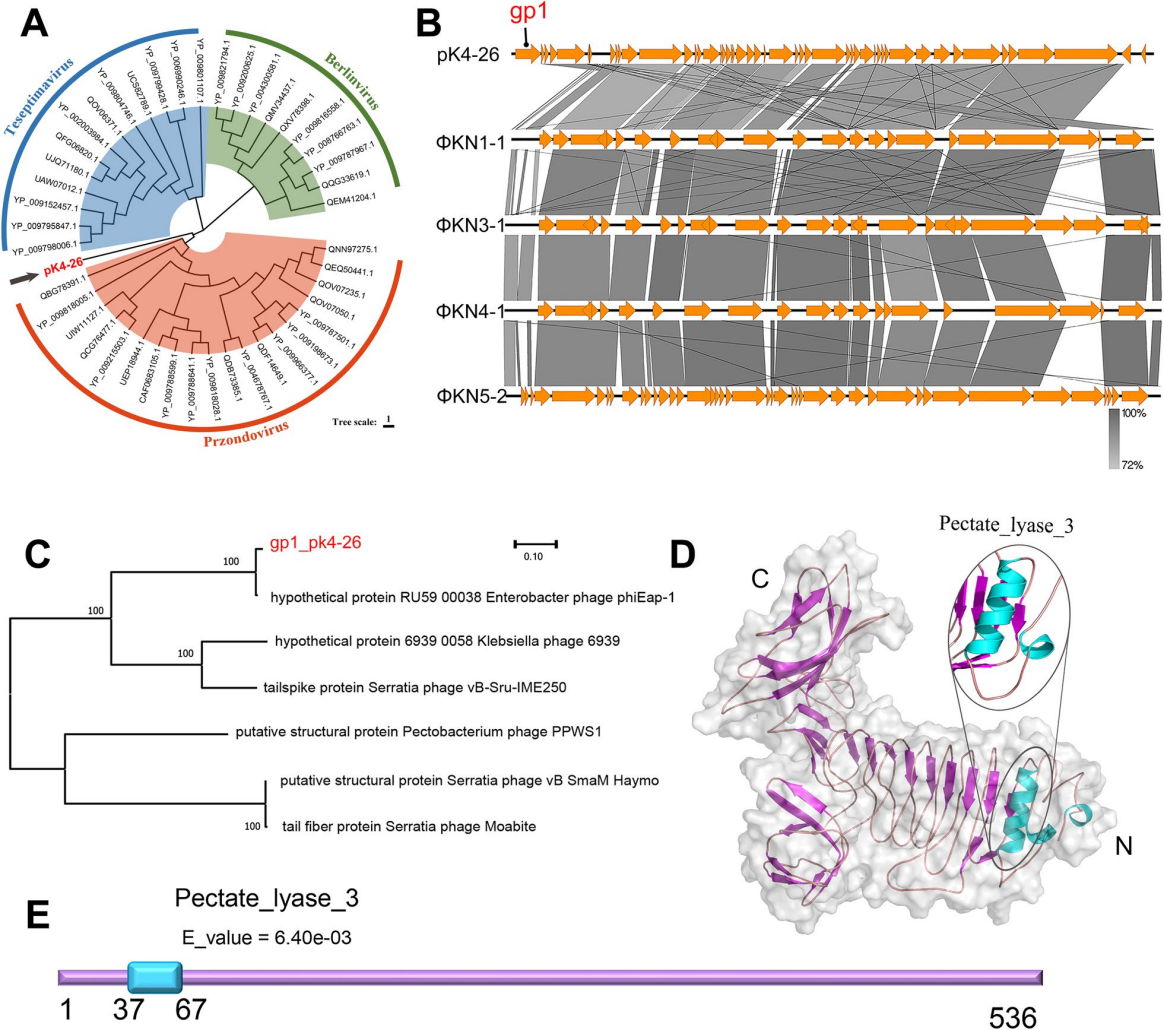
Bioinformatics analysis of phage pK4-26 and the putative depolymerase. **A** Phylogenetic tree based on amino acid sequences of the large terminase subunit using maximum likelihood method with 1000 bootstrap replicates. **B** Comparative genomic analysis. **C** Phylogenetic tree based on gene sequences of the putative depolymerase using maximum likelihood method with 1000 bootstrap replicates. **D** Protein structure prediction of the putative depolymerase. **E** Protein domain prediction of the putative depolymerase

To clarify the unique characteristics of the phage, its genome was compared to those of other phages in public databases. The phylogenetic tree showed that phage pK4-26 was clustered with the genus *Przondovirus* and was on a separate branch (Fig. 3A). It has a similar genetic structure to those of phage KN3-1, phage KN1-1 (NC_048129.1), phage KN4-1 (NC_048130.1), and phage K5-2 (NC_047798.1) (Fig. 3B), and the unmatched regions in the pK4-26 genome are the first CDS (*gp1*). Very few possible proteins are homologous to this sequence in the NCBI database. Phylogenetic analysis (Fig. 3A) showed that the protein was divided into one branch with the hypothetical protein RU59_00038 (YP_009196380.1) of the *Enterobacter* phage phiEap-1, which infects *Enterobacter aerogenes* (*K. aerogenes*). None of the other homologous proteins on different branches were derived from phages that infect *K. aerogenes*, which suggests that the protein possibly has host specificity. Other homologous proteins were annotated as structural proteins or tailspike proteins, so the protein may be a structural phage protein. Moreover, the protein contains a Pectate_lyase_3 domain in the N-terminal region, which was preset as a parallel β-helix fold structure (Fig. 3D, E).

### A novel capsule depolymerase was found and can degrade biofilms

**Fig. 4:**
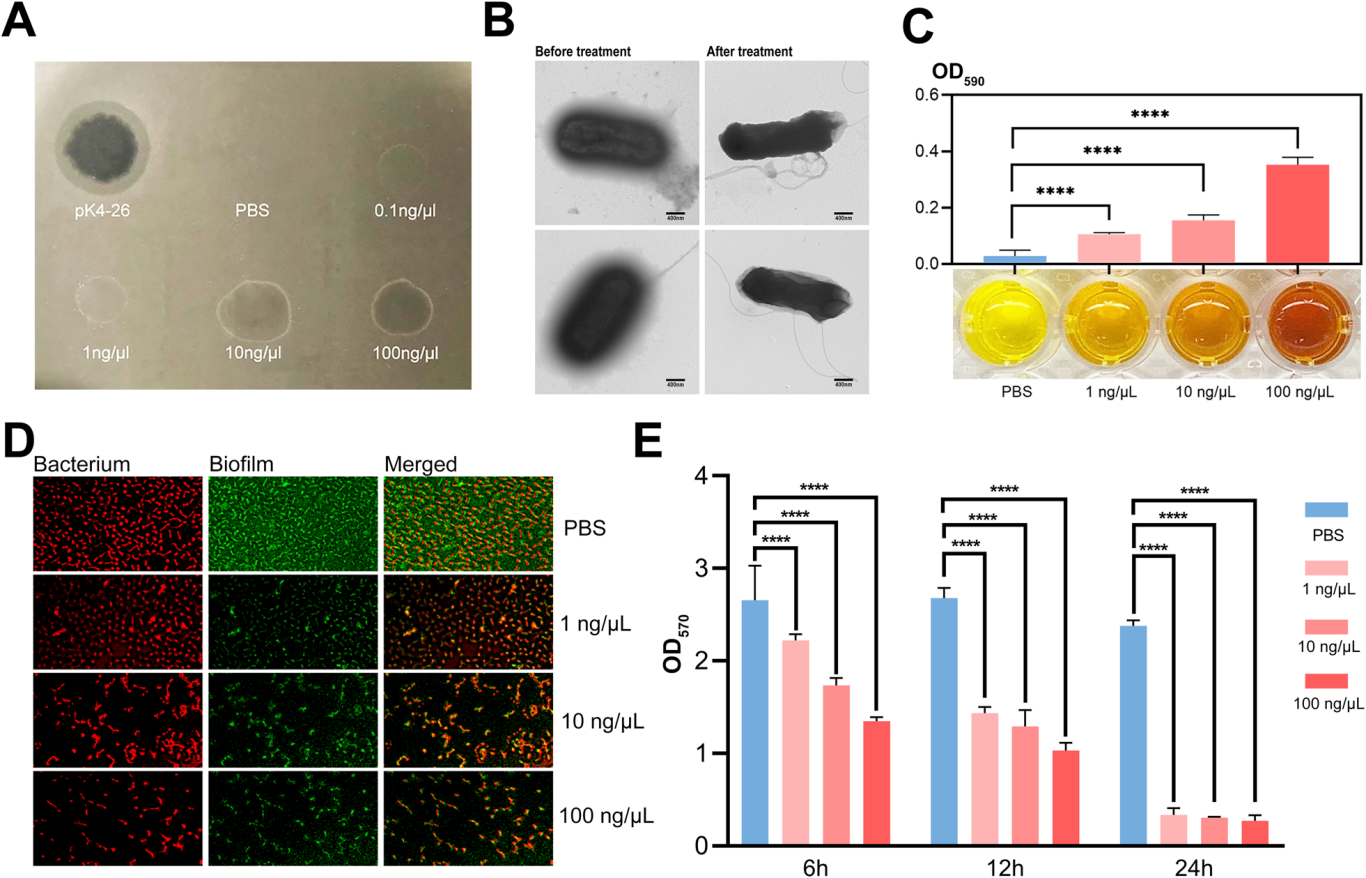
*K4-26 dep* protein can digest capsules and degrade biofilms. **A** The translucent plaques formed by the pK4-26 and *K4-26 dep* protein. **B** Morphology of *K. aerogenes* 4-26 under TEM before and after treatment using depolymerase. **C** Color change in DNS reacted with *K. aerogenes* 4-26 CPS. **D** Fluorescence staining showed that the depolymerase can significantly reduce the amount of biofilm formed by *K. aerogenes* 4-26. **E** Crystal violet staining also proved the *K4-26 dep* protein can degrade biofilm formed by *K. aerogenes* 4-26. Values are expressed as mean ± SD (n ≥ 3). ****P < 0.0001 (one-way ANOVA)

The putative gene (*gp1*) was cloned and expressed via a pET-28a expression system. The protein formed translucent plaques in a dose-dependent manner and had a similar host range to phage pK4-26. Interestingly, all hosts belonged to the PSgc4 type (Fig. 4A, Table 1). This protein made the capsule around the bacteria disappear, making the surface of the bacteria smooth to rough. It degraded the extracted CPS into reducing sugars (Fig. 4B, C) Thus, the protein was a novel capsule depolymerase. Furthermore, the depolymerase significantly reduced biofilms formed by *K. aerogenes* 4-26 in both time-dependent and dose-dependent manners, thereby reducing the number of bacteria attached to the biofilm (Fig. 4D, E). The results showed that 1 ng/μL *K4-26 dep* protein eliminated more than 80% of biofilms in 24 h.

### *K4-26 dep* protein can reduce virulence, and improve sensitivity to innate immunity

**Fig. 5:**
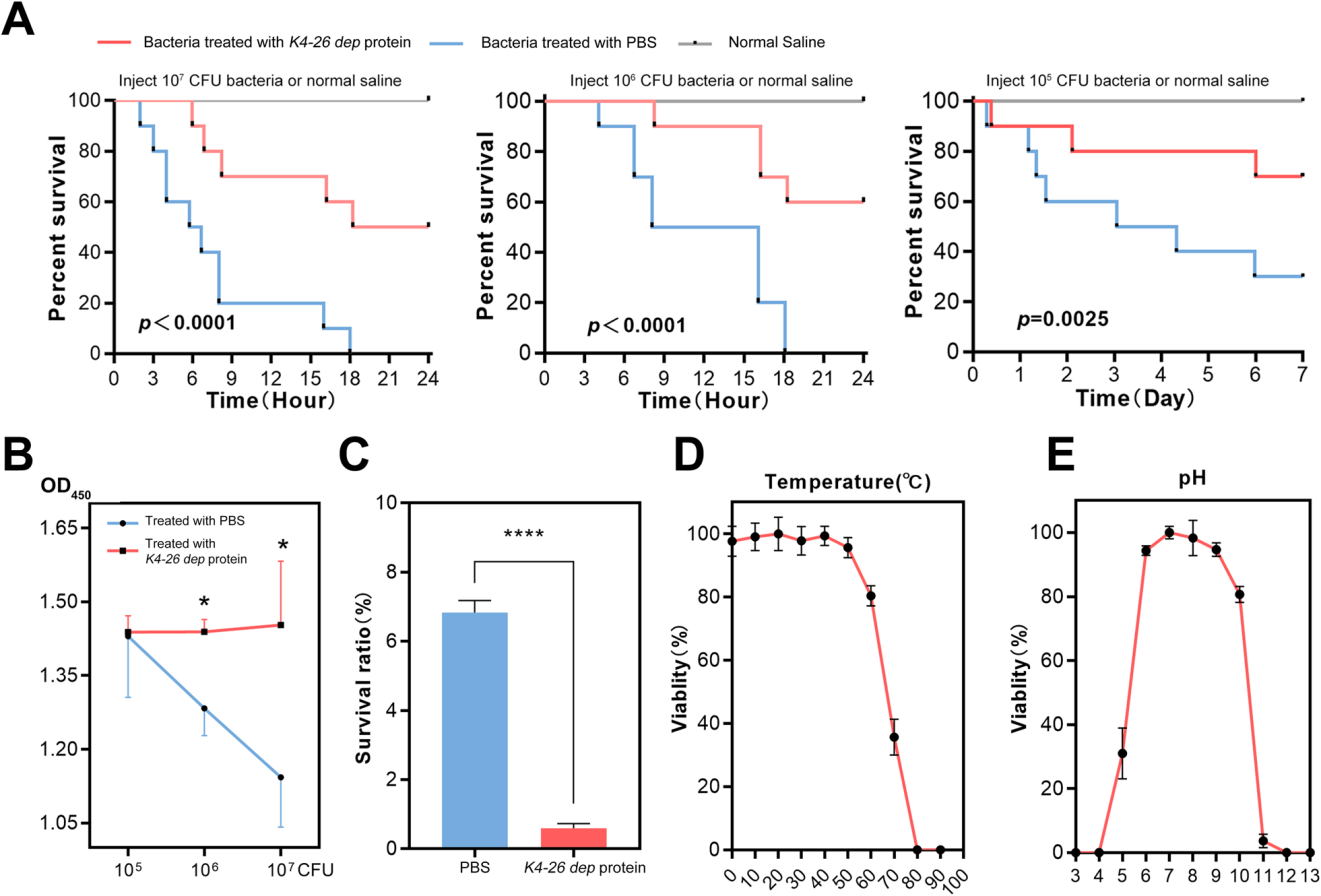
Functional analysis and characterization of *K4-26 dep* protein. **A** Survival curve of *G. mellonella* larvae under different treatments (Kruskal–Wallis’s test). **B** CCK-8 assay reflecting bacterial virulence under different treatments. **C** The survival of depolymerase-treated bacteria by serum killing. **D** Thermal stability of phage. **E** pH stability of phage. Values are expressed as mean ± SD (n ≥ 3). *P < 0.05; ****P < 0.0001 (one-way ANOVA)

The capsule is one of the sources of bacterial virulence, and its presence is associated with phagocytosis resistance and increased mortality in animal models. After incubation with the depolymerase, *K. aerogenes* 4-26 caused less cell death and lower death rate of in *G. mellonella* larvae (Fig. 5A, B). The depolymerase also improved the sensitivity of *K. aerogenes* 4-26 to innate immunity (Fig. 5C). Moreover, the *K4-26 dep* protein remained stable in environments below 60 °C (Fig. 5D) and at a pH between 6 and 10 (Fig. 5E), thus showing slightly higher thermal stability than phage pK4-26.

## Discussion

*Klebsiella aerogenes* is a member of the ESKAPE group, which is the most frequently isolated species in clinical infections, especially in immunocompromised patients and those hospitalized in intensive care units. It is frequently associated with a multidrug resistance phenotype [27]. Phages are a promising tool that can be used to address the absence of a drug in the post-antibiotic era. Phages have several advantages over antibiotics including strain specificity, lack of structural destruction of the intestinal microbiome and low development costs [28]. Therefore, finding new phages, establishing phage banks, and studying the interaction mechanisms between phages and host bacteria is of great significance.

In recent years, several studies have reported on phages that target *K. aerogenes*; however, these studies were limited to phage biology and genomic characteristics [23, 29–31]. There is a lack of kinetic research on clinical treatment with phages, and our understanding of the interaction mechanisms between bacteriophages and bacteria needs to be deepened. This study isolated a novel phage targeting *K. aerogenes* named pK4-26 from sewage. It is a new member of the *Przondovirus* genus. By closely monitoring bacterial and phage abundance over time in animal models, we demonstrated that the phage could replicate itself and lyse *K. aerogenes *in vivo, reducing mortality and alleviating inflammation.

Phages of the *Podoviridae* family often exhibit depolymerases. These enzymes can bind to the host bacteria’s CPS, exopolysaccharides, or lipopolysaccharides and specifically cleave polysaccharide repeat units [18]. Clear and round plaques with a halo are a sign that the phages carry depolymerases. The phage pK4-26 also formed plaques with a halo, so we speculated that pK4-26 may carry a depolymerase.

Phages of the *Przondovirus* genus have a similar genomic structure [32, 33]. In 2019, Pan et al. identified three *Podoviruses* infecting *Klebsiella* that encoded different capsule depolymerases digesting specific capsule types [32]. We compared the genetic structure of pK4-26 with those phages and found an unmatched region. Therefore, we suspected that the unmatched region (*gp1*) may encode a depolymerase.

Generally, depolymerases encoded by phages consist of two parts, one has depolymerization function; the other is annotated as a structural protein [34]. This protein was predicted to have a Pectate_lyase_3 domain. Phylogenetic analysis showed that the protein was divided into one branch with the hypothetical protein RU59_00038 (YP_009196380.1) of the *Enterobacter* phage phiEap-1, which showed highly similarity (identity: 97.95%, query coverage: 100%). None of the other homologous proteins were derived from phages that infect *K. aerogenes*, which suggests that the protein possibly has host specificity. Other homologous proteins were annotated as structural or tailspike proteins, so the protein may be a structural phage protein.

There are few publications relating to depolymerases of *K. aerogenes* phage. We downloaded 3879 protein sequences of 24 phages that infect *K. aerogenes* from the NCBI viral genome database and analyzed them to find special domains such as Pectate_lyase_3, Hydrolase, Endo-*N*-acetylneuraminidases, and Glycanase [35]. We discovered that there were few proteins with depolymerization functions (Table 2, Additional file 3: Table S5). The hypothetical protein RU59_00038 (YP_009196380.1) of *Enterobacter* phage phiEap-1 has a Pectate_lyase_3 domain. *Klebsiella* phage N1M2 has a putative structural lysozyme (QGH72084.1) containing a Glyco_hydro_19 domain that is a member of a family of glycoside hydrolases, and the protein may function as a lysozyme [36] or depolymerase [37]. The phage is known to be able to remove a preformed *K. aerogenes* biofilm [31]. vB_KaeM_KaOmega has a putative cell wall hydrolase containing a hydrolase_2 domain that may have lysozyme or depolymerase function [35]. The small number of these predicted proteins may be due to limited research on phages targeting *K. aerogenes*, highlighting our research’s importance.

**Table 2:** Mining potential depolymerases from NCBI viral genomes database

Scientific name	SEQ_ID	Hmm name	Type	E_value
*Enterobacter* phage phiEap-1	YP_009196380.1	Pectate_lyase_3	Family	0.00089
*Klebsiella* phage N1M2	QGH72084.1	Glyco_hydro_19	Domain	3.60E−06
*Klebsiella* phage vB_KaeM_KaOmega	QEG12163.1	Hydrolase_2	Family	1.3e−22

The host range of *Klebsiella* phages and their extended depolymerases is thought to be associated with serotypes based on capsule features [38–41]. However, there is still no accepted standard for serotyping *K. aerogenes*. In 2019, Guo et al. established a molecular serotyping scheme based on surface polysaccharide antigens [42], and following the method, the hosts of pK4-26 and the *K4-26 dep* protein all belong to PSgc4. This means that they may be serotype specific. The PSgc4-type stains accounted for approximately 33% of clinical isolates, which means that pK4-26 and depolymerase may have a wider-spectrum killing power against clinical strains.

Furthermore, we discovered that the protein can efficiently degrade biofilms formed by *K. aerogenes* 4-26. However, when *K4-26 dep* protein was added in advance, biofilm formation was not inhibited, but enhanced (data not presented). This may be because the bacteria degraded the protein and used it to form thicker, tighter biofilms [43].

Considering that the capsule is an important virulence factor, we explored the effect of depolymerase on bacterial virulence and immune sensitivity. After treatment with the depolymerase, the virulence of *K. aerogenes* 4-26 to both animal and cell models was reduced. After losing the capsule, the immune evasion ability of *K. aerogenes* 4-26 was also reduced and its sensitivity to innate immunity improved. We then tested the temperature stability of the depolymerase and found that it had slightly higher temperature resistance than pK4-26.

Many studies have confirmed that phages have good efficacy and safety in treating pneumonia and intestinal inflammation caused by *Klebsiella* in animal models [44, 45]. In addition, the depolymerases derived from *Klebsiella* phages have been proven to have the ability to digest capsules, remove biofilms, and reduce bacterial virulence [46, 47], and they have potential uses such as reducing lethality, vaccine development and clinical strain typing [38, 48, 49]. More exploration of phage pK4-26 and its depolymerases is worth studying.

## Conclusions

To our knowledge, this is the first phage kinetic study targeting *K. aerogenes.* Our data demonstrated that pK4-26 has good efficacy and safety for treating *K. aerogenes* infections in mouse models. It carries a depolymerase that removes biofilms, reduces virulence, and improves intrinsic immune sensitivity. Indeed, more preclinical studies and mechanistic studies are needed for clinical use.

### Supplementary Information

**Additional file 1: Figure S1.** H&E staining of liver, kidney, and spleen in different groups at 24 h and 48 h.

**Additional file 2: Figure S2.** Phage susceptibility testing of bacteria. (A) Description of strain source. (B) Phage susceptibility testing of bacteria isolated from the lungs at 7 days in the treatment group.

**Additional file 3: Table S1.** Primer sequences and PCR procedure used for prokaryotic expression. **Table S2.** The minimum inhibitory concentration (MIC) values of the bacteria mentioned. **Table S3.** Comparison of pK4-26 with other phage genome sequences in the NCBI database. **Table S4.** The putative open reading frames of phage pK4-26. **Table S5.** Protein domain prediction of *Klebsiella aerogenes* phage.

## Data Availability

All data generated or analyzed during this study are included in this published article and its Additional files.
