# Supplementary figures and images for: A novel phage carrying capsule depolymerase effectively relieves pneumonia caused by multidrug-resistant Klebsiella aerogenes
Source: J Biomed Sci. 2023 Aug 31;30:75. doi: 10.1186/s12929-023-00946-y (PMC10470133; doi:10.1186/s12929-023-00946-y)

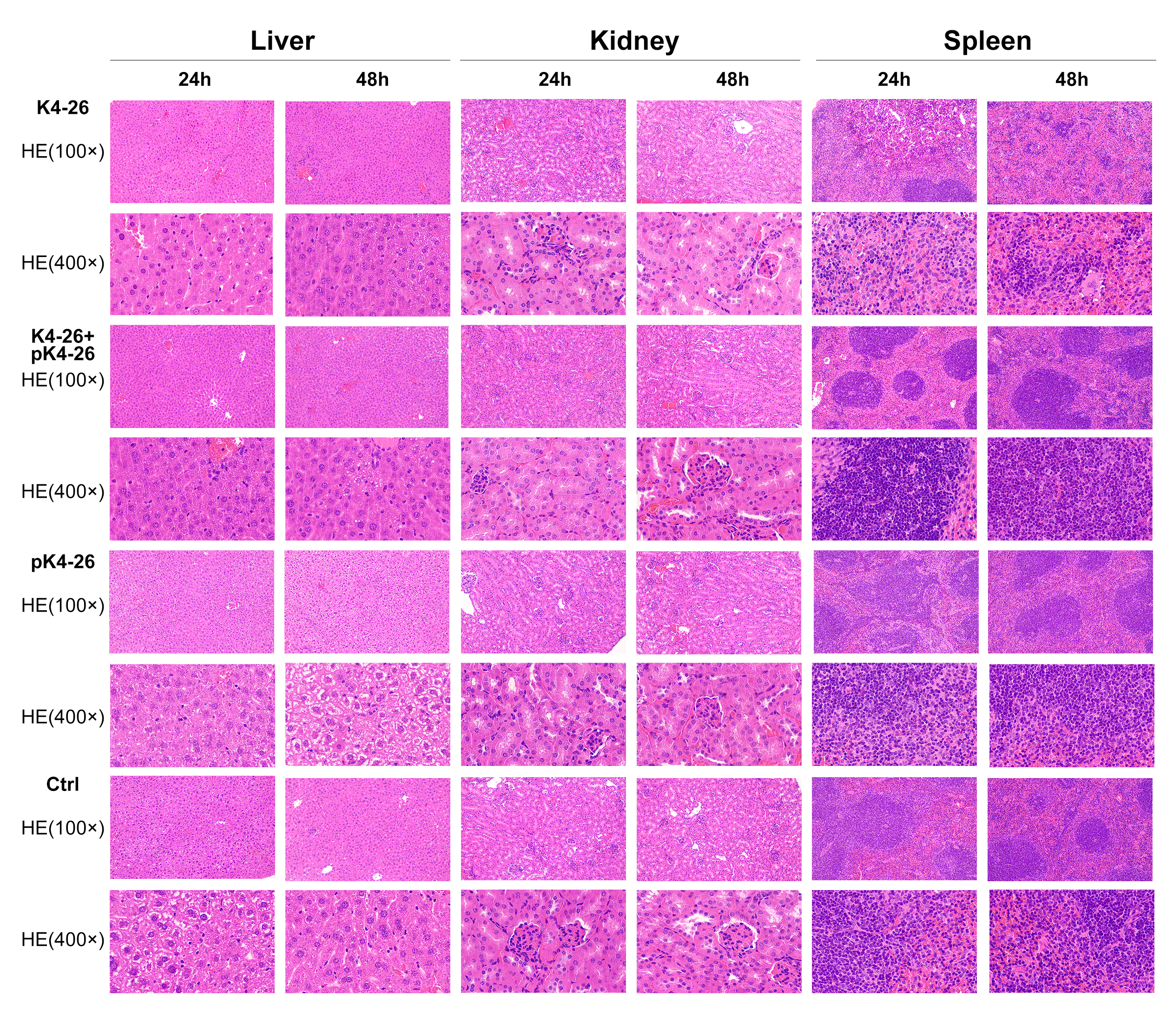

Supplement: Supplementary file 1 — Additional file 1: Figure S1. H&E staining of liver, kidney, and spleen in different groups at 24 h and 48 h. [file 12929_2023_946_MOESM1_ESM.tif]

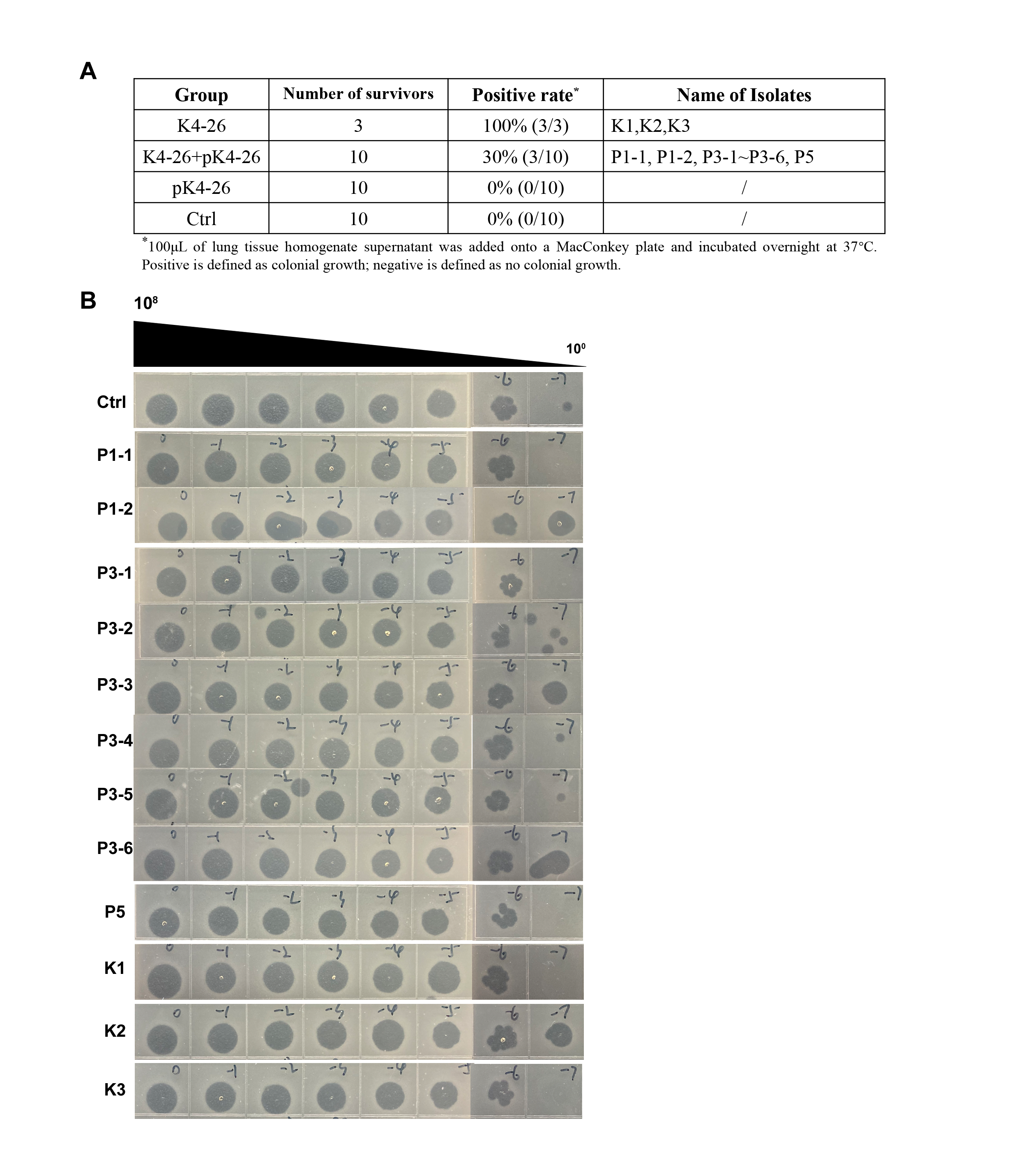

Supplement: Supplementary file 2 — Additional file 2: Figure S2. Phage susceptibility testing of bacteria. (A) Description of strain source. (B) Phage susceptibility testing of bacteria isolated from the lungs at 7 days in the treatment group. [file 12929_2023_946_MOESM2_ESM.tif]
